# The Impact of Categorical Proximity on Positive and Negative Priming: Evidence from Categorisation and Naming Tasks

**DOI:** 10.3390/bs16060906

**Published:** 2026-06-03

**Authors:** Diana Chopchik, Margarita Filippova

**Affiliations:** N. P. Bechtereva Institute of the Human Brain Russian Academy of Sciences (IHB RAS), 9 Akademika Pavlova Str., St. Petersburg 197376, Russia; filippova@ihb.spb.ru

**Keywords:** negative priming, positive priming, categorical proximity, inhibition, episodic retrieval

## Abstract

This study compares the breadth and temporal dynamics of positive and negative priming effects depending on the depth of stimulus processing. Two experiments were conducted using a Naming task (shallow processing) and a semantic Categorisation task into “living/non-living” (deep processing) with varying categorical proximity of the prime and probe. A dissociation between the patterns of positive and negative priming was observed. Positive priming emerged exclusively in the Categorisation task, demonstrated graded sensitivity to categorical proximity, and increased with longer response times, consistent with spreading activation models. Negative priming, by contrast, was observed in both tasks: in the Naming task, it appeared as a stable effect independent of categorical proximity, whereas in the Categorisation task, it manifested as a short-lived effect restricted to fast responses. These findings support the early automatic inhibition account and contradict the predictions of episodic retrieval models. Overall, the results indicate functional differences between positive and negative priming under the present conditions: whereas positive priming appears to implement fine-grained semantic tuning, negative priming serves as a coarse, early inhibitory mechanism operating at the level of generalised representations.

## 1. Introduction

### 1.1. Positive and Negative Priming

The repeated presentation of a stimulus alters the speed and accuracy of its subsequent processing: at times, repetition facilitates the response (positive priming, PP), while at others, it decelerates it (negative priming, NP). If a stimulus has previously been the focus of attention, its subsequent recognition is accelerated—an instance of the PP effect. Conversely, if a stimulus was previously ignored, its subsequent recognition is slowed—giving rise to the NP effect (Tipper, 1985; Neill et al., 1992).

PP reflects a process of spreading activation within the semantic network, which is well-established in the literature (Collins & Loftus, 1975; Neely, 1991). According to the spreading activation theory (Collins & Loftus, 1975), the presentation of a word (e.g., DOG) activates not only its corresponding node but also related nodes (e.g., CAT), thereby facilitating the subsequent recognition of these words. It has been demonstrated that the PP effect is more pronounced when the semantic similarity between prime and probe is higher (e.g., Lucas, 2000; Falikman & Koifman, 2005).

In contrast, the picture for NP is less clear. Whereas semantic PP is considered a relatively stable and reproducible effect observable across various stimulus types and tasks (e.g., naming, lexical decision, categorisation), semantic NP presents as an exceedingly fragile and unreliable phenomenon. It appears highly sensitive to specific paradigm details and processing demands (e.g., Fox, 1995; Neill & Kahan, 1999; Chao & Yeh, 2008; Frings et al., 2015; Megías et al., 2020). Indeed, a multitude of studies have failed to detect semantic NP altogether (e.g., Chiappe & Macleod, 1995; Catena et al., 2002).

This inconsistency can be differentially accounted for by the two dominant theories of NP. The first—inhibition theory—proposes that during the prime display, the ignored distractor is actively suppressed. A residual inhibition then impedes the subsequent activation of the same or a related representation during the probe (Tipper, 1985; Houghton & Tipper, 1994). According to this view, an NP effect emerges only if the distractor is initially activated and then successfully inhibited. The second—episodic retrieval theory—interprets NP as a consequence of the probe stimulus retrieving a prior memory episode in which it was tagged as ‘to-be-ignored’. The retrieval of this ‘ignore’ tag creates response conflict, manifesting as response slowing (Neill & Valdes, 1992; Neill et al., 1992). This model posits that for NP to occur, a sufficiently integrated memory trace of the prime episode—linking the stimulus, its context, and the required response—must be formed and later retrieved (otherwise, its retrieval is impossible).

### 1.2. Negative Priming: Inhibition vs. Episodic Retrieval

It should be noted that contemporary models distinguish between automatic and controlled inhibition (Frings et al., 2015). The former is an early-stage process arising from shallow prime processing and suppresses the distractor’s representation; the latter is a slower process associated with motor response blocking and conflict resolution at the decision-making stage (Ortells et al., 2016; Megías et al., 2020). In the present work, the term ‘inhibition’ refers specifically to the automatic suppression of representations.

#### 1.2.1. Temporal Parameters

The proposed mechanisms of inhibition and episodic retrieval imply distinct temporal dynamics. The episodic retrieval model describes NP as a consequence of re-accessing a previously formed memory episode containing a ‘to-be-ignored’ tag; this process requires time to reinstate the bindings between the stimulus, its context, and the previous response (Neill & Valdes, 1992; Tipper & Driver, 1988). Consequently, the effect magnitude of NP attributable to this mechanism should increase with longer response times, as completing the retrieval process demands additional time. According to May et al. (1995), episodic retrieval accounts imply stronger NP effects at later stages of processing, when reinstatement of prior stimulus–response episodes becomes more likely.

Conversely, inhibition is construed as a rapid, automatic mechanism triggered within a narrow temporal window, often prior to stimulus identification, and serves to selectively suppress irrelevant stimulus representations or interpretations (Neely, 1991; Houghton & Tipper, 1994; Fox, 1995). Such a mechanism should be evident even on the fastest reactions, as it operates independently of slower, controlled processing stages (Neill & Valdes, 1992; Houghton & Tipper, 1994; Fox, 1995; May et al., 1995; Grison & Strayer, 2001; Tse et al., 2011).

#### 1.2.2. Prime–Probe Similarity

Another critical distinction between inhibition and episodic retrieval mechanisms concerns the breadth of NP generalisation. Historically, the very possibility of semantic NP was considered evidence against the episodic retrieval account, as this theory initially presupposed that the prime and probe episodes must match. Consequently, NP effects were expected to be confined to identical stimuli (Neill & Valdes, 1992; Chiappe & Macleod, 1995; Neill & Kahan, 1999). Although modern hybrid models allow for NP with only partial feature overlap (Catena et al., 2002; Neill & Joordens, 2002; Chao & Yeh, 2008; Frings et al., 2015), episodic retrieval accounts still assume that effect magnitude depends on the similarity between the retrieved memory episode and the current stimulus. Therefore, NP driven by this mechanism should be maximal for identical stimuli and diminish as their similarity decreases.

In contrast, inhibition theory, positing the suppression of central semantic representations, predicts that NP can generalise not only to identical stimuli but also to semantically related ones (Tipper, 1985; Fox, 1995; Damian, 2000; Frings & Wentura, 2006). Thus, the pattern of NP as a function of semantic similarity between prime and probe may reveal the predominant underlying mechanism: a gradient decrease in the effect with increasing semantic distance aligns with the episodic retrieval model, whereas the inhibition account permits a broader range of semantic generalisation.

#### 1.2.3. Depth of Prime Processing

According to the episodic retrieval model, NP arises when a memory trace of a previously ignored stimulus, tagged as ‘to-be-ignored’, is retrieved. It is posited that deeper processing of the prime facilitates the formation of a more distinct episodic trace, thereby increasing the likelihood of its subsequent retrieval (Neill & Valdes, 1992; May et al., 1995; Frings et al., 2015). Consequently, tasks requiring meaningful stimulus analysis—such as semantic categorisation or matching based on semantic features—create conditions more favourable to NPs driven by episodic retrieval (Mayr & Buchner, 2007; Chao & Yeh, 2008).

In contrast, tasks focused on rapid responding without obligatory access to meaning—such as naming (Tipper, 1985; Damian, 2000), naming with colour-based selection (Schrobsdorff et al., 2012), or perceptual matching (Caudek & Domini, 2013; Labossière & Leboe-McGowan, 2018)—may prevent the prime from reaching a level of conscious semantic representation, thus reducing the probability of binding between prime and probe episodes. Conversely, inhibition theory holds that distractor suppression is possible at early processing stages, prior to semantic awareness, and does not require deep analysis (Tipper, 1985; Houghton & Tipper, 1994; Fox, 1995; Grison & Strayer, 2001; Tse et al., 2011). Thus, variations in the depth of prime processing, as dictated by task type, can also influence which mechanism predominantly underlies the NP effect.

In summary, the distinction between inhibitory and episodic retrieval processes is primarily reflected in the degree of prime–probe similarity, response time, and depth of prime processing (Table 1).

### 1.3. Breadth of Positive and Negative Priming Effects

As noted previously, it is well-established that the magnitude of PP increases reliably with greater semantic similarity between prime and probe (Lucas, 2000; Falikman & Koifman, 2005), a finding consistent with the spreading activation model (Collins & Loftus, 1975). For NP, the findings are more mixed. The breadth of negative priming generalisation remains debated. Some studies report that NP is confined to identical or highly similar stimuli (Chiappe & Macleod, 1995; Damian, 2000), supporting episodic retrieval models. Others demonstrate NP generalisation across semantic categories, perceptual features, or even task contexts (Neumann et al., 1999; Leboe et al., 2005; Caudek & Domini, 2013; Filippova et al., 2025), which aligns with an inhibition account. These contradictory findings continue to motivate further research.

Overall, despite the extensive literature, the question of whether NP spreads through the semantic network in the same manner as PP remains open. Contradictory findings may reflect differences in experimental conditions that modulate the relative contribution of inhibitory and episodic retrieval processes (May et al., 1995). Key parameters governing this balance are the degree of prime–probe similarity, the depth of prime processing, and temporal response characteristics. However, there is a lack of studies in which these parameters are systematically varied within a single experimental design.

In a broader context, a fundamental question concerns whether target activation and distractor inhibition constitute independent mechanisms of information selection (Christou et al., 2025; Marko et al., 2026) and whether PP and NP can be isolated within a single experimental paradigm (Burnham, 2026).

### 1.4. The Present Study

The present study addresses this gap by comparing the magnitude of PP and NP effects in both Naming and Categorisation tasks across four levels of categorical proximity between prime and probe, while also accounting for response time. This design enables the concurrent evaluation of:

- The sensitivity of PP and NP to semantic similarity;
- The influence of prime processing depth (Naming vs. Categorisation);
- The temporal dynamics of these effects within a task.

Research Hypotheses:

If NP primarily reflects an episodic retrieval mechanism, then:

1. The effect magnitude will be maximal for identical stimuli and diminish with decreasing semantic similarity;
2. The effect will increase with longer response times;
3. The effect magnitude will be greater in the Categorisation task, which permits deeper processing, compared to the Naming task.

If NP primarily reflects an automatic inhibition mechanism, then:

1. The effect will not depend on the degree of semantic similarity within a category, demonstrating a broad equivalence range;
2. The effect will be observed even on the fastest responses, consistent with automatic processes;
3. The effect magnitude will be greater in the Naming task, which does not require deep semantic analysis, compared to the Categorisation task.

The statistical analyses were designed to test three key predictions derived from inhibition and episodic retrieval models: (1) the influence of semantic similarity—using rmANOVA, planned comparisons, and Spearman correlations; (2) the influence of processing depth—through comparison of priming patterns in the Naming and Categorisation tasks; and (3) the influence of response time—using linear mixed-effects models in which participants were compared across response speed groups.

## 2. Methods

### 2.1. Experiment 1

This experiment investigated whether negative priming (NP) under conditions of shallow processing (i.e., a Naming task) demonstrates broad semantic generalisation—that is, independence from categorical distance—and manifests at early response latencies, which would align with predictions of the automatic inhibition model. It also examined whether positive priming (PP) under the same conditions exhibits the expected gradient pattern dependent on categorical distance.

#### 2.1.1. Participants

Fifty-two adults participated in the study (34 females, 18 males), aged between 18 and 58 years (M = 29.08, SD = 10.43). The sample included both university students (30.76%) and adults with completed higher education (69.24%). All participants were native Russian speakers with normal or corrected-to-normal vision. Prior to inclusion, all participants completed a structured screening questionnaire covering demographic characteristics, native language, vision status, education level, presence/absence of neurological or psychiatric disorders, and current acute medical conditions. Participants reporting uncorrected vision, neurological or psychiatric disorders, or acute illness were excluded from participation.

An a priori power analysis for a 2 (condition: NP vs. control) × 4 (categorical proximity) repeated-measures ANOVA was conducted using G*Power (Version 3.1) to evaluate the interaction effect. Based on previous studies employing similar paradigms (Tipper & Cranston, 1985; Damian, 2000; Marí-Beffa et al., 2005), semantic NP effects were expected to fall within the range of approximately 20–40 ms (η^2^_p_ ≈ 0.10; f ≈ 0.30). The analysis was conducted with α = 0.05 and power (1–β) = 0.80, yielding a minimum required sample size of 36 participants.

The study was approved by the Ethics Committee of N.P. Bekhtereva Institute of the Human Brain of the Russian Academy of Sciences (approval no. 2, dated 20 February 2025) and conducted in accordance with the Declaration of Helsinki. All participants provided informed consent prior to participation.

#### 2.1.2. Materials and Design

A standard semantic priming paradigm with a Naming task was employed, requiring participants to name target words while ignoring distractors. The stimuli comprised 16 four-letter Russian words from the categories ‘living’ (wolf, tiger, duck, owl, rose, peony, linden, maple) and ‘non-living’ (chair, wardrobe, coat, soap, needle, weight, umbrella, sieve).

Categorical proximity between prime and probe was manipulated across four levels, ranging from identity to broad categorical membership. Non-living stimuli were used in the control condition to ensure the absence of semantic similarity between prime and probe: both the prime target and distractor belonged to the non-living category, whereas the probe target was always living in experimental trials. The word frequency for ‘living’ and ‘non-living’ categories did not differ statistically, *t*(14) = −0.332, *p* = 0.74. All stimuli were controlled for visual orthographic neighbourhood (Coltheart’s N). No orthographic neighbours differing by a single letter were present within the stimulus set (Coltheart’s *N* = 0 for all words).

It is important to note that the same stimulus words were rotated across levels of categorical proximity within participants and across experimental conditions between participants; therefore, the lexical characteristics of individual words were automatically balanced within the experimental design.

Examples of proximity levels:

1. Level 1: duck–duck, identical words (10 trials per stimulus list);
2. Level 2: duck–owl, both are birds (10 trials per stimulus list);
3. Level 3: duck–wolf, both are animals (20 trials per stimulus list);
4. Level 4: duck–rose, both are ‘living’ (40 trials per stimulus list);
5. Control: duck–wardrobe, no semantic relation.

The experiment employed a within-subjects design with three conditions, defined by the relationship between the probe target and the preceding prime display:

1. Attended Repetition (AR): The target of the probe was identical or related to the attended target of the preceding prime;
2. Ignored Repetition (IR): The target of the probe was identical or related to the ignored distractor of the preceding prime;
3. Control: The target of the probe was unrelated to both the target and the distractor of the preceding prime.

On each trial, a target (red word) and a distractor (green word) were presented simultaneously. The colours were chosen to provide high perceptual contrast and followed standard practices used in negative priming paradigms (e.g., Tipper, 1985; Rosner et al., 2015; Kristjánsson et al., 2002). Participants were instructed to name only the red target aloud while ignoring the green distractor.

Each participant completed 128 experimental trials (AR, IR, Control), distributed across the four levels of categorical proximity. An additional 32 filler trials with ‘non-living’ targets were included to maintain task variability and unpredictability. In total, each participant completed 170 trials, including 32 filler trials and 10 practice trials. The study lasted approximately 30–35 min, including a short break in the middle.

Stimulus lists were counterbalanced across participants such that each word appeared in only one condition for a given participant but was rotated through conditions between participants. Due to the limited stimulus set and the counterbalancing procedure, the exact number of trials per proximity level and conditions varied slightly between stimulus lists. Thus, in List 1: NP = 32, PP = 48, Control = 48, Fillers = 32; List 2: NP = 48, PP = 48, Control = 32, Fillers = 32; List 3: NP = 48, PP = 32, Control = 48, Fillers = 32. However, across the entire sample, the design was fully balanced: each word rotated through all conditions, and each participant received an equal total number of trials. The trial order was fully randomised for each participant, ensuring that the three experimental conditions were evenly distributed within each participant’s session.

Only reaction times (RTs) to target stimuli in probe trials were included in the analysis. Responses from prime trials and filler trials were excluded.

#### 2.1.3. Procedure

**Figure 1:**
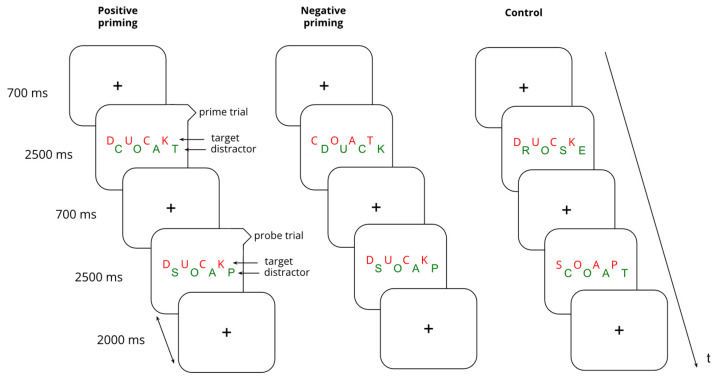
Stimulus Sequence for Each Condition. Note: The large arrow indicates the temporal order of events within a trial. Small arrows between displays indicate the response-stimulus interval (RSI). Arrows pointing to the words identify the target and distractor stimuli. Target words are shown in red and distractor words in green.

Each trial commenced with a fixation cross displayed for 700 ms, followed by the prime and probe displays. Stimuli were presented until a keypress or for a maximum of 2500 ms. Participants were instructed to name the red word aloud as quickly as possible and then press the spacebar to proceed to the next trial. The interval between the prime and probe displays (RSI) was 700 ms. The inter-trial interval was 2000 ms. The trial sequence is illustrated in Figure 1.

The response–stimulus interval (RSI = 700 ms) was selected based on an analysis of previous studies employing similar experimental paradigms. This interval falls within the range (500–900 ms) in which the literature has documented manifestations of both automatic inhibition effects (Tipper & Cranston, 1985; Marí-Beffa et al., 2005) and episodic retrieval effects (Chiappe & Macleod, 1995; Damian, 2000).

Speech responses were recorded via headset microphones and analysed using Praat software, version 6.4.20 (Boersma & Weenink, 1992/2022). Speech onset was defined as the first clear increase in acoustic waveform amplitude confirmed by corresponding changes in the spectrogram, excluding preceding non-speech sounds. This approach remains the gold standard in psycholinguistic naming tasks because it allows reliable differentiation of true speech onset from non-speech artefacts (breaths, lip smacks, microphone noise, or hesitations). Trials containing artefacts in the speech recording or exhibiting incorrect timing (e.g., a button press preceding speech onset) were excluded from analysis because such trials did not allow reliable estimation of speech-onset latency. The same exclusion criteria were applied uniformly across all participants and experimental conditions.

#### 2.1.4. Statistical Procedures

Statistical analyses were conducted in R (version 4.5.2).

Trials with RTs exceeding ±2 SD from the group mean and trials containing audio recording artefacts were removed. In total, 12.19% of trials were excluded: 8.17% due to recording artefacts or unreliable speech-onset detection identified during manual inspection in Praat, and 4.01% due to RT trimming (±2 SD criterion). In the Naming task, participants did not produce incorrect responses; exclusions were therefore limited to technical artefacts associated with voice-response recording.

Mean RTs per condition were submitted to a two-way repeated-measures ANOVA (2 × 4 rmANOVA) with factors Condition (Attended repetition, Ignored repetition) and Categorical Proximity (4 levels). The control condition was not included as a separate level in the rmANOVA because it served as a common baseline for estimating priming effects rather than as a level of categorical proximity. Paired-sample *t*-tests were used for planned comparisons between each proximity level and the control condition, as the theoretical predictions allowed for local manifestations of priming effects. Spearman’s rank correlations were computed between RT and proximity level to assess whether priming effects changed monotonically as a function of categorical proximity.

To test whether the magnitude of negative priming increases with reaction time (as predicted by episodic retrieval accounts) or emerges early in processing (as expected under inhibitory accounts), a temporal dynamics analysis was conducted. Participants were divided into three RT-based groups (terciles: fast (≤607 ms, *n* = 17), medium (608–685 ms, *n* = 18), and slow (≥686 ms, *n* = 17)). RT distributional approaches are commonly used in priming research to investigate temporal dynamics that may remain hidden when analysing only mean RTs (Balota et al., 2008; Tse et al., 2011). As noted by Balota et al. (2008), “changes in mean performance (or the lack thereof) may reflect distinct patterns at the level of underlying RT distributions” (p. 459).

Separate linear mixed-effects models were estimated for AR and IR conditions because these conditions were associated with different theoretical predictions regarding the temporal dynamics of priming effects. The models included fixed effects of Condition, RT Group, and their interaction. Because the design involved multiple repeated measurements for each participant and item, random intercepts for participants and items were included in the models. In addition, random slopes for Condition by participants were included to account for potential individual differences in the magnitude of priming effects across experimental conditions.

### 2.2. Experiment 2

According to the theoretical distinction between inhibition and episodic retrieval mechanisms, tasks requiring deeper semantic processing should favour episodic retrieval processes. To test this prediction, Experiment 2 employed a semantic Categorisation task (‘living’/‘non-living’), which requires explicit access to word meaning. This task creates conditions conducive to slower, more controlled processes, including the formation and subsequent retrieval of memory episodes. Experiment 2 investigated whether NP effects become stronger with increasing categorical proximity between prime and probe, and whether they increase with longer response times, as predicted by the episodic retrieval model.

Furthermore, it examined whether the PP effect in this task demonstrates the predicted gradient pattern dependent on categorical proximity.

#### 2.2.1. Participants

Forty volunteers aged 18 to 48 years participated in the study (31 females, 9 males; M = 24.7, SD = 5.6). Participants included university students (35%) and individuals with completed higher education (65%). All participants were native Russian speakers with normal or corrected-to-normal vision. The screening procedure and exclusion criteria were identical to those used in Experiment 1. None of the participants had taken part in Experiment 1.

#### 2.2.2. Materials and Design

The materials and design for Experiment 2 were identical to those used in Experiment 1. The same set of 16 four-letter Russian nouns from the ‘living’ and ‘non-living’ categories was employed, and the four-level manipulation of categorical proximity between prime and probe was retained. The overall experimental design replicated that of the first experiment (see Section 2.1 for details), with the critical change being the use of a semantic Categorisation task (‘living’/’non-living’) instead of Naming.

However, the control condition was adapted for the Categorisation task. Preliminary analyses indicated that classifying non-living targets was generally slower than classifying living targets (analysis of prime targets: 746.7 ms vs. 714.4 ms; *t*(39) = 3.90, *p* < 0.001). To avoid this systematic bias, the condition with the maximum categorical distance (the fourth proximity level, e.g., duck—rose) was used as the control in this experiment. At this level, there was no significant difference between the AR and IR conditions (729 ms vs. 746 ms; *t*(39) = −1.26, *p* = 0.216), justifying its use as a neutral baseline.

#### 2.2.3. Procedure

The experimental procedure was identical to that of Experiment 1 (see Section 2.1.3 and Figure 1). The only procedural modification concerned the method of response. Participants were instructed to classify the red target word as quickly and accurately as possible by pressing the corresponding key on the computer keyboard (‘left arrow’ for ‘living’, ‘right arrow’ for ‘non-living’), while ignoring the green distractor.

#### 2.2.4. Statistical Procedures

Trials with errors, timeouts, or reaction times exceeding two standard deviations from the individual participant’s mean were excluded from analysis. In total, 4.59% of trials were discarded.

Mean RTs per condition were submitted to a two-way repeated-measures ANOVA (2 × 3 rmANOVA) with factors Condition (Attended repetition, Ignored repetition) and Categorical Proximity (3 levels). The control condition was not included as a separate level in the rmANOVA because it served as a common baseline for estimating priming effects rather than as a level of categorical proximity. Paired-sample *t*-tests were used for planned comparisons between each proximity level and the control condition, as the theoretical predictions allowed for local manifestations of priming effects. Spearman’s rank correlations were computed between RT and proximity level to assess whether priming effects changed monotonically as a function of categorical proximity.

Similar to Experiment 1, RT distributional analyses were conducted to assess the temporal dynamics of priming effects across fast, medium, and slow responders. Threshold values were 647 ms (lower tercile) and 718 ms (upper tercile), defining groups of fast (*n* = 13), medium (*n* = 14), and slow (*n* = 13) participants. Linear mixed-effects models were estimated separately for AR and IR conditions, with fixed effects of Condition, RT Group, and their interaction; random intercepts for participants and items, as well as random slopes for Condition by participants, were included in the models.

## 3. Results

### 3.1. Experiment 1

#### 3.1.1. Effects of Categorical Proximity

Neither the main effect of condition (*F*(1, 46) = 0.015, *p* = 0.90, *η*^2^ < 0.001), nor the main effect of categorical proximity (*F*(1, 46) = 1.20, *p* = 0.31, *η*^2^ = 0.026), nor their interaction (*F*(3, 138) = 0.67, *p* = 0.57, *η*^2^ = 0.014) reached significance. At the same time, robust NP effects were observed in both the IR and AR conditions (Table 2).

No significant correlation between RT and categorical proximity was found in either the AR (*ρ*(119) = 0.044, *p* = 0.53) or IR (*ρ*(119) = 0.023, *p* = 0.74) condition.

#### 3.1.2. Temporal Dynamics Analysis

**Figure 2:**
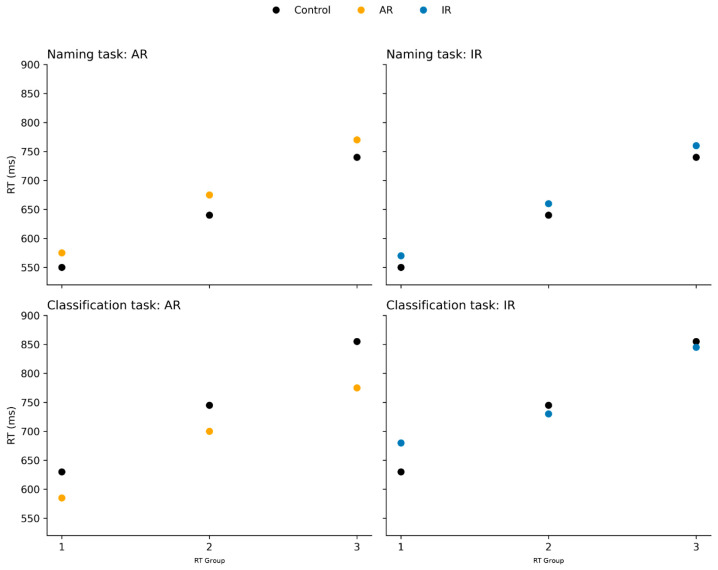
Mean reaction times (RTs) in the attended repetition (AR), ignored repetition (IR), and control conditions, by RT group (fast, medium, slow).

Attended Repetition. A significant main effect of condition was observed (χ^2^(1) = 6.011, *p* = 0.014). The Condition × RT Group interaction was not significant (χ^2^(2) = 1.254, *p* = 0.534). As illustrated in Figure 2, a visually comparable NP effect was evident across all reaction speed groups: for fast (−24.8 ms), middle (−38.3 ms), and slow (−32.8 ms) RT groups. Pairwise comparisons (Control vs. AR) revealed statistically significant differences for the middle (*t*(24.1) = −2.596, *p* = 0.016) and slow (*t*(25.4) = −2.188, *p* = 0.038) RT groups; for the fast RT group, the difference did not reach significance (*t*(24.4) = −1.673, *p* = 0.107).

Ignored Repetition. A significant main effect of condition was found (χ^2^(1) = 4.949, *p* = 0.026). The Condition × RT Group interaction was not significant (χ^2^(2) = 2.914, *p* = 0.233). Visually comparable NP effects were again observed for the fast (−24.2 ms), middle (−19.4 ms), and slow (−35.7 ms) RT groups (Figure 2). Pairwise comparisons (Control vs. IR) yielded a statistically significant difference only for the slow RT group (*t*(21.1) = −2.710, *p* = 0.013). Differences for the fast (*t*(20.4) = −1.862, *p* = 0.077) and middle (*t*(20.4) = −1.498, *p* = 0.150) RT groups were not significant.

Experiment 1 demonstrated that under conditions of shallow processing (i.e., the Naming task), NP exhibited characteristics consistent with an automatic inhibition mechanism: the effect was independent of semantic distance and did not show a clear, systematic increase in magnitude with longer RTs (despite the NP effect in both AR and IR conditions reaching statistical significance primarily for participants with ‘slow’ responses, the interaction between condition and RT group was not significant).

### 3.2. Experiment 2

#### 3.2.1. Effects of Categorical Proximity

A significant main effect of Condition (*F*(1, 37) = 30.79, *p* < 0.001, *η*^2^ = 0.454) and a significant Condition × Categorical Proximity interaction (*F*(2, 74) = 5.9, *p* = 0.004, *η*^2^ = 0.137) were found. The main effect of Categorical Proximity was not significant (*F*(2, 74) = 2.01, *p* = 0.141, *η*^2^ = 0.023). Robust PP effects were observed in the AR condition, while no significant NP was found in the IR condition (Table 3).

Furthermore, a significant positive correlation was found between categorical proximity and RT in the AR condition (*ρ*(119) = 0.244, *p* = 0.008), confirming that the positive priming effect decreased as semantic distance increased. No such correlation was observed in the IR condition (*ρ*(119) = −0.043, *p* = 0.545).

#### 3.2.2. Temporal Dynamics Analysis

Attended Repetition. A significant main effect of condition was found (*χ*^2^(1) = 18.126, *p* < 0.001). The Condition × RT Group interaction did not reach significance (*χ*^2^(2) = 2.050, *p* = 0.359). However, as shown in Figure 2, the positive priming effect was largest for the slow RT group: 42.2 ms for the fast group (*t*(35.0) = 1.780, *p* = 0.084), 46.3 ms for the middle group (*t*(35.4) = 2.051, *p* = 0.048), and 85.7 ms for the slow group (*t*(37.7) = 3.570, *p* = 0.001).

Ignored Repetition. In contrast, for the IR condition, the main effect of Condition was not significant (*χ*^2^(1) = 0.52, *p* = 0.473). However, a significant Condition × RT Group interaction was identified (*χ*^2^(2) = 7.39, *p* = 0.025), indicating that the effect differed across response speed groups. A negative priming effect was observed only in the fast RT group, at −51.11 ms (*t*(35.9) = −2.629, *p* = 0.013). No significant difference from the control condition was found for the middle or slow RT groups: 15.30 ms (*t*(33.8) = 0.850, *p* = 0.401) and 8.44 ms (*t*(36.4) = 0.436, *p* = 0.665), respectively. These results are clearly illustrated in Figure 2.

Experiment 2 demonstrated that under conditions of deeper semantic processing (i.e., the Categorisation task), PP showed the predicted gradient pattern, decreasing with increasing semantic distance and becoming most pronounced among ‘slow’ responders. In contrast, no overall NP effect was observed in the IR condition; however, a significant NP effect emerged selectively in the fast RT group.

## 4. General Discussion

The present study compared the breadth and temporal dynamics of positive priming (PP) and negative priming (NP) effects under conditions of shallow stimulus processing (i.e., a Naming task) and deeper semantic processing (i.e., a Categorisation task). Previous theoretical and empirical accounts suggest that NP may arise from both automatic inhibition and episodic retrieval processes, with the relative contribution of these mechanisms depending on semantic similarity, processing depth, and temporal dynamics (Neill & Valdes, 1992; May et al., 1995; Neill & Kahan, 1999; Damian, 2000; Frings et al., 2015). Accordingly, the present study examined whether NP would demonstrate a narrow similarity-dependent pattern increasing with ‘slow’ responses, as predicted by episodic retrieval accounts, or a broader and more rapid pattern consistent with inhibition-based accounts. A distinctive feature of the present study was the direct comparison of PP and NP breadth under identical experimental conditions, allowing us to examine whether the two effects spread through semantic space in a similar manner. The results demonstrated a clear dissociation between PP and NP patterns.

The categorical proximity-dependent PP effect observed in the Categorisation task aligns precisely with predictions derived from spreading activation models (Collins & Loftus, 1975; Lucas, 2000). Furthermore, the PP effect was most pronounced among ‘slow’ responders. In contrast, the NP effect was independent of categorical proximity and did not increase with longer response times. This challenges the role of episodic retrieval as the primary mechanism for NP under the employed conditions and tasks. The findings are more consistent with early automatic inhibition accounts, whose characteristics, however, differ depending on the task type.

Experiment 1 (Naming Task). A key finding was the observation of a robust NP effect, which was already present among fast responders and showed no dependence on the degree of categorical proximity between prime and probe. This lack of a proximity-dependent gradient contradicts predictions of the episodic retrieval model, which posits maximal NP for identical stimuli with a weakening effect as similarity decreases (Neill & Valdes, 1992; Neill & Joordens, 2002). Instead, the observed pattern suggests that inhibition was applied to a generalised representation at the level of the ‘living’ category. This suggests that NP can operate at a representational level common to a whole group of semantically related stimuli. Similar patterns have been reported in studies demonstrating NP generalisation across semantic categories, perceptual features, and task contexts. For example, Labossière and Leboe-McGowan (2018) showed that NP can arise based on semantic-category membership rather than exact stimulus matching, whereas PP remained sensitive only to complete prime–probe overlap. Likewise, Neumann et al. (1999) found that NP persisted across language changes in bilingual participants under conditions where PP dissipated. Leboe et al. (2005) demonstrated that NP not only persisted but also increased following a task switch, whereas PP was substantially reduced. Schrobsdorff et al. (2012) reported that NP, unlike PP, remained stable despite changes in stimulus colour. Caudek and Domini (2013) found that NP generalised both within and between semantic categories, whereas PP was confined to within-category associations. Finally, Filippova et al. (2025) demonstrated NP generalisation to an entire category of previously learned prime-pseudowords, suggesting suppression of broadly “uninformative” representations rather than specific stimulus identities. Collectively, these findings support the view that NP may operate at relatively broad representational levels.

An unexpected result was the presence of NP not only in the Ignored Repetition condition but also in the Attended Repetition condition. This may indicate that, within the context of a Naming task employing a limited set of frequently repeated stimuli, semantic activation itself can create interference that requires suppression for efficient access to lexical form. Within inhibition frameworks, this could be interpreted as the suppression of ‘within-category noise’ (preventing excessive spread of activation within a category) rather than suppression of the specific stimulus. This aligns with contemporary views of NP not solely as a consequence of distractor ignoring but also as a by-product of selection and representational stabilisation processes (Frings et al., 2015).

Experiment 2 (Categorisation Task). In this task, the PP effect was strongest among ‘slow’ responders. This suggests that in a Categorisation task, PP is not purely automatic; its magnitude increased with time, indicating a significant contribution from controlled processes. In contrast to the robust PP, no significant overall NP effect was found for the entire sample. However, an analysis based on response speed revealed a significant NP effect among fast responders. This indicates that even within a task requiring deeper processing, automatic inhibition of the ignored distractor can still be implemented during the initial stages of stimulus processing. The transient nature of NP under deep processing conditions has been noted previously in the literature (e.g., Fox, 1995). Our data provide further support for this pattern.

The present results are difficult to reconcile with episodic retrieval accounts. If NPs in the Categorisation task were related to the retrieval of the prime episode, they should have increased with longer response times (Fox, 1995; Tse et al., 2011). Conversely, the obtained data fit the predictions of automatic inhibition models (Tipper & Cranston, 1985; Houghton & Tipper, 1994), which posit that distractor suppression manifests before controlled processes (e.g., response-level conflict resolution) become dominant. In our view, the ignored stimulus is suppressed before the completion of its semantic analysis, at a stage where it has not yet been differentiated to the level of a specific meaning. Such a mechanism is functionally justified: for rapid distractor ignoring, it is advantageous for the cognitive system to block an entire potentially relevant region of semantic space rather than selectively suppressing each individual item. This explains why, on the fastest responses—where decisions are based predominantly on automatic processes—the residual inhibition from the ignored distractor has time to manifest. For ‘slow’ responders, controlled strategic processes intervene, potentially overwriting or masking the early inhibition effect.

Although the present findings are more consistent with inhibition-based accounts, hybrid models cannot be fully excluded, since previous studies have also reported NP patterns that are more readily explained by episodic retrieval mechanisms (in particular, NP observed under conditions involving exact stimulus repetitions and deeper semantic processing). For example, Chiappe and MacLeod (1995) observed reliable NP for identical words across both Naming and Categorisation tasks, whereas semantically related stimuli failed to produce NP. Similarly, Damian (2000) reported NP for identical pictures across tasks, while semantic NP emerged only in the Categorisation task. Such findings suggest that the relative contribution of inhibition and episodic retrieval may shift depending on task parameters. From this perspective, the present findings may be compatible with both inhibition accounts and with hybrid approaches proposing that the relative contribution of inhibition and episodic retrieval varies across processing conditions (May et al., 1995; Neill & Kahan, 1999; Frings et al., 2015).

The results of the present study suggest that under the current experimental conditions, PP reflects a fine-tuning of the semantic network, whereas NP may reflect the operation of a coarse but rapid ‘reset’ mechanism affecting an entire category, operating across a broad semantic range.

### Limitations

Several limitations of the study should be noted. First, our study did not analyse the full set of prime–probe relations (e.g., distractor → distractor, target → distractor, role-reversal conditions) recommended by Christie and Klein (2008) for negative priming research. According to these authors, the use of a limited set of conditions may make it difficult to rule out alternative explanations. Therefore, future studies incorporating the complete set of prime–probe relations could provide a more rigorous test of competing explanations of NP.

Second, as noted by May et al. (1995), the relative contribution of inhibition and episodic retrieval may depend on the response–stimulus interval (RSI). In the present study, the RSI was not manipulated; a fixed interval was used, at which both mechanisms have previously been observed to operate. Systematic manipulation of the RSI in future research could further elucidate the temporal dynamics of NP.

Third, sample sizes differed between the two experiments, which may limit the precision of direct cross-experimental comparisons.

Finally, although we interpreted NP in the naming task as a manifestation of automatic inhibition, we cannot entirely rule out a contribution from episodic retrieval. Hybrid models (Catena et al., 2002; Neill & Joordens, 2002; Chao & Yeh, 2008; Frings et al., 2015) suggest that both mechanisms may operate in parallel. Therefore, the obtained results only allow us to conclude that, under the present experimental conditions, the pattern of NP is more consistent with inhibition-based theories.

## 5. Conclusions

The present study demonstrates a fundamental distinction between negative priming and positive priming effects. PP, observed during deep semantic processing of the prime (i.e., the Categorisation task), exhibits the classic signature of spreading activation: its magnitude depends on categorical proximity and increases over time. In contrast, the observed NP patterns—specifically, their independence from categorical distance and the absence of a systematic increase with longer response times—are more consistent with automatic inhibition accounts than with explanations based primarily on episodic retrieval under the present experimental conditions.

A key finding is the demonstration of the context-dependent nature of this inhibition. A robust NP effect, independent of semantic similarity under conditions of shallow processing (i.e., the Naming task), stands in contrast to a transient NP effect observed only during the initial stages of stimulus processing under conditions of deep semantic analysis (i.e., the Categorisation task).

## Figures and Tables

**Table 1:** Conditions for Inhibition vs. Episodic Retrieval Manifestation.

Parameter/Condition	Episodic Retrieval	Inhibition	Sources
Depth of prime processing	Deep processing is necessary for binding the prime and probe episodes.	Shallow processing suffices; inhibition can be triggered automatically.	(Tipper & Cranston, 1985; Neely, 1991; Neill & Valdes, 1992; Fox, 1995; May et al., 1995; Frings et al., 2015)
Prime–probe similarity	NP likelihood increases with greater similarity (especially identity).	NP is less dependent on similarity, as inhibition can occur at the level of more abstract categories.	(Neill & Joordens, 2002; Mayr & Buchner, 2007; Rothermund et al., 2005)
Response time	NP magnitude increases with longer RTs; the effect accumulates as more time is available for retrieval.	The effect is present even (or sometimes exclusively) on the fastest reactions.	(Neill & Valdes, 1992; Houghton & Tipper, 1994; May et al., 1995; Grison & Strayer, 2001; Tse et al., 2011)

**Table 2:** Mean RTs, Priming Effects, and Paired *t*-Test Results by Condition and Categorical Proximity in the Naming Task.

Condition	Proximity Level	RT (M ± SD), ms	Control RT (M ± SD), ms	NP Effect (ms)	*t*	*p*
AR	1	659.6 (111.8)	637.7 (83.7)	−21.9	2.28	0.027
	2	677.4 (100.9)	634.8 (82.1)	−42.6	4.38	<0.001
	3	666.4 (94.7)	637.5 (83.7)	−28.9	4.29	<0.001
	4	673.0 (93.3)	639.5 (84.0)	−33.5	5.32	<0.001
IR	1	671.1 (109.7)	639.5 (84.0)	−31.6	2.99	0.004
	2	675.5 (103.9)	639.8 (81.5)	−35.6	4.22	<0.001
	3	661.3 (91.8)	637.5 (83.7)	−23.8	4.54	<0.001
	4	665.9 (95.4)	639.5 (84.0)	−26.4	5.02	<0.001

Note: AR = Attended Repetition; IR = Ignored Repetition. RT = Reaction Time. Negative values indicate slower responses in the experimental condition relative to control (NP effect).

**Table 3:** Mean RTs, Priming Effects, and Paired *t*-Test Results by Condition and Categorical Proximity in the Categorisation Task.

Condition	Proximity Level	RT (M ± SD), ms	Control RT (M ± SD), ms	NP Effect (ms)	*t*	*p*
AR	1	640.3 (122.0)	750.5 (102.2)	+110.2	5.53	<0.001
	2	689.6 (143.4)	738.0 (128.3)	+48.4	2.43	0.020
	3	704.0 (135.3)	738.0 (128.3)	+34.0	4.29	0.023
IR	1	763.3 (169.6)	735.8 (129.3)	−27.4	1.04	0.307
	2	767.5 (157.8)	738.0 (128.0)	−29.6	1.16	0.253
	3	735.3 (116.6)	738.0 (128.0)	+2.6	0.16	0.870

Note: AR = Attended Repetition; IR = Ignored Repetition. RT = Reaction Time. Positive values indicate faster responses (PP effect). Negative values indicate ‘slow’ responses in the experimental condition relative to control (NP effect).

## Data Availability

The data presented in this study are openly available in the Open Science Framework (OSF) repository at https://osf.io/b8mer/ (accessed on 20 May 2026).

## Abbreviations

The following abbreviations are used in this manuscript:

PP	Positive priming
NP	Negative priming
AR	Attended repetition
IR	Ignored repetition
